# Comparative efficacy of locally delivered 1.2% rosuvastatin and 1.2% simvastatin gel as an adjunct to non-surgical periodontal therapy in chronic periodontitis: a clinical and microbiological assay

**DOI:** 10.1007/s44445-026-00239-8

**Published:** 2026-09-23

**Authors:** Haripriya Rajaram, G. Hima Bindu, K. Rekha Rani, Mehul A. Shah, Vignesh Kamath

**Affiliations:** 1 Department of Periodontology and Implantology, Malla Reddy Institute of Dental Sciences, Malla Reddy Vishwavidyapeeth (Deemed to be University), Suraram, Hyderabad, Telangana 500055 India; 2 Department of Periodontology and Implantology, Panineeya Mahavidyalaya Institute of Dental Sciences and Research Centre, Hyderabad, India; 3 Department of Public Health Dentistry, Government Dental College and Hospital, Chhatrapati Sambhajinagar (Aurangabad), Maharashtra, India; 4 Department of Prosthodontics Crown and Bridge, Manipal College of Dental Sciences Mangalore, Manipal Academy of Higher Education, Manipal, India

**Keywords:** Chronic periodontitis, Local drug delivery, Rosuvastatin, Simvastatin, Polymerase chain reaction.

## Abstract

To evaluate and compare the clinical and microbiological efficacy of locally administered 1.2% Rosuvastatin (RSV) gel and 1.2% Simvastatin (SMV) gel as an adjunct to scaling and root planing (SRP) in the treatment of patients with chronic periodontitis. In this exploratory comparative study, 60 qualifying periodontal pockets (probing pocket depth [PPD] of 5–7 mm) from patients aged 25–55 years were randomized into three groups ($n = 20$ pockets per group): Group I received SRP + local delivery of 1.2% RSV gel; Group II received SRP + local delivery of 1.2% SMV gel; and Group III (control) received SRP + placebo gel. Clinical parameters—Plaque Index (PI), Modified Gingival Index (MGI), Sulcus Bleeding Index (SBI), PPD, and Clinical Attachment Loss (CAL)—were evaluated at baseline, 6 weeks, and 3 months. Microbiological assessment of *Porphyromonas gingivalis (Pg)* and *Aggregatibacter actinomycetemcomitans (Aa)* was performed using Polymerase Chain Reaction (PCR) at baseline and 6 weeks. All 60 sites completed the study with no dropouts or adverse reactions. At 6 weeks and 3 months, both statin groups (Groups I and II) demonstrated statistically significant reductions in MGI, SBI, PPD, and CAL compared to the placebo group ($P < 0.05$). The statin groups also showed significantly lower levels of Aa and Pg at 6 weeks. Comparison between the two statins revealed that the SMV group achieved greater reductions in clinical indices (MGI, SBI, PPD, CAL) and bacterial counts (Aa and Pg) than the RSV group; however, this difference was not statistically significant (P < 0.05). No significant differences in PI scores were found between any of the groups at any interval (P < 0.05). The local delivery of both 1.2% simvastatin gel and 1.2% rosuvastatin gel as adjuncts to non-surgical periodontal therapy significantly improved clinical and microbiological outcomes in chronic periodontitis. While simvastatin demonstrated slightly greater absolute improvements than rosuvastatin, the overall therapeutic difference between the two statins was not statistically significant.

## Introduction

Periodontitis is an infectious and inflammatory condition characterized by the destruction of the soft and hard tissues of the periodontium, which can eventually lead to tooth loss (Kinane and Stathopoulou 2017). The microorganisms that inhabit the subgingival region are believed to be the cause of inflammation and destruction that take place near the bone; among them, *Aggregatibacter actinomycetemcomitans* (*Aa*) and *Porphyromonas gingivalis* (*Pg*) are known to be major pathogens in the onset and development of periodontitis (Elavarasu et al. 2012). 

The goal of current periodontal treatment is to eliminate bacterial accumulations from the tooth surface and to shift the harmful microbiota toward a state that supports periodontal health (Elavarasu et al. 2012). Scaling and root planing (SRP) is considered the most reliable method; however, this procedure by itself may not fully eradicate the suspected microorganisms from the pocket due to infiltration of these organisms into deeper regions that are not reachable by periodontal instruments, leading to periodontal recurrence (Agarwal et al. 2016). Therefore, various adjunctive pharmacological therapies have been explored to enhance the efficacy of SRP.

Systemically administered drugs for the treatment of periodontitis have several drawbacks that can be overcome by applying the drug locally. These approaches enable a reduction in total administered dosage, thereby minimizing adverse effects while maintaining therapeutic amounts of the drug in the gingival crevicular fluid (GCF) for a longer period and, consequently, longer maintenance of the therapeutic effects (Agarwal et al. 2016). 

Statins are a category of drugs that block the HMG-CoA reductase enzyme, mainly prescribed to manage high blood lipid levels and prevent heart disease (Martin-Ruiz et al. 2018). Beyond their ability to lower cholesterol, statins offer a broad spectrum of additional health benefits—including anti-inflammatory, antioxidant, antimicrobial (bacterial, fungal, and viral), and immunomodulatory effects—while also actively promoting bone growth (Catherine Petit et al. 2019). 

As a naturally derived statin older than its counterparts, simvastatin (SMV) possesses lipophilic properties and has proven to be anti-inflammatory and antimicrobial against various organisms (Kamińska Petit et al. 2019, Emani Petit et al. 2014). Rosuvastatin (RSV), a newer, synthetic, second-generation, sulfur-containing, hydrophilic statin, has been shown to have good antihyperlipidemic and strong anti-inflammatory activity. Methylcellulose is a safe, hypoallergenic, and non-irritating polymer that can be used as a controlled-release vehicle in many oral and topical medicinal products in the mouth (Pradeep et al. 2015). 

This study aimed to assess and compare the effectiveness of the locally administered 1.2% Rosuvastatin gel and Simvastatin gel along with scaling and root planing.

Studies have shown that local drug delivery (LDD) of 1.2% RSV is effective in improving clinical and radiographic outcomes compared to 1.2% atorvastatin (ATV) or placebo gels when used alongside mechanical periodontal therapy. Research has also indicated that LDD of 1.2% ATV produces better periodontal results than 1.2% SMV (Pradeep et al. 2016, Martande et al. 2017). However, no study has evaluated the microbiological efficacy of 1.2% RSV and 1.2% SMV against *Pg* and *Aa*. The present study aims to compare the clinical and microbiological effectiveness of 1.2% RSV gel and 1.2% SMV gel in the treatment of periodontal disease.

## Materials and methods

### Study design

A prospective, three-arm, parallel-group, double-blinded, randomized, placebo-controlled clinical trial.

### Study population and source of data

The study population comprised patients diagnosed with chronic periodontitis who attended the Outpatient Department (OPD) of the Panineeya Mahavidyalaya Institute of Dental Sciences and Research Centre in Dilsukhnagar, Hyderabad. Before recruitment, all eligible participants were comprehensively briefed on the nature and objectives of the research, and written informed consent was voluntarily obtained from each individual.

The sample size was calculated a priori using the primary outcome variable of change in probing pocket depth (PPD). Based on data from a previous randomized controlled trial evaluating locally delivered statin gel as an adjunct to scaling and root planing, an expected mean difference (∆) and standard deviation (σ) were considered clinically significant. Assuming a two-sided significance level (α) of 0.05 and a study power (1 − β) of 80%, the required sample size was calculated using the following formula for comparison of means:

$$[n=\frac{2,(Z_{\alpha/2}+Z_\beta)^2\sigma^2}{\Delta^2}]$$

where:

(n) = sample size per group.

(Z_{α/2}=1.96) (95% confidence interval).

(Z_ β= 0.84) (80% power).

(Σ) = expected standard deviation.

(∆) = minimum clinically important difference.

The calculated minimum sample size was **20 periodontal sites per group**. Therefore, a total of **60 periodontal sites** equally allocated to three study groups were included.

(Charan J, Biswas T. *How to calculate sample size for different study designs in medical research?* Indian Journal of Psychological Medicine. 2013;35(2):121–126.)

### Method of collection of data

The participants in the study were informed about the research, and written consent was acquired.

- The study sample consisted of a total of 60 periodontal pockets categorized into 3 groups, each group comprised of 20 sites.

o Group I (20): Scaling and Root Planing (SRP) with application of 1.2% Rosuvastatin gel.

o Group II (20): SRP along with application of 1.2% Simvastatin gel.

o Group III (20): SRP along with application of placebo gel.

### Inclusion criteria

- Patients aged between 25 to 55years.
- Probing pocket depth (PPD) 5–7 mm.
- Patients who have not received periodontal therapy within the preceding 6 months.

### Exclusion criteria

- Patients who received treatment with antibiotics/anti-inflammatory or any statin drugs in the last 3 months.
- Pregnant and lactating females.
- Medically compromised patients.
- Smokers, tobacco chewers and alcoholics.
- Patients who were allergic to drugs.
- Severe form of Periodontitis with deep pockets.

### Clinical and microbiological outcomes

Clinical parameters were meticulously evaluated at baseline, 6 weeks, and 3 months post-intervention. These assessments included the:

- Plaque Index (PI) according to Silness and Löe (1964).
- Modified Gingival Index (MGI) by Lobene et al. (1986)
- Sulcus Bleeding Index (SBI) as described by Mühlemann and Son (1971).
- Probing Pocket Depth (PPD).
- Clinical Attachment Loss (CAL).

In addition to the clinical parameters, a microbiological assessment was conducted using Polymerase Chain Reaction at baseline and at 6 weeks to evaluate the levels of *Porphyromonas gingivalis* and *Aggregatibacter actinomycetemcomitans* using Cycle Threshold values (Ct).

### Formulation of 1.2% rosuvastatin gel and 1.2% simvastatin gel

**Fig. 1:**
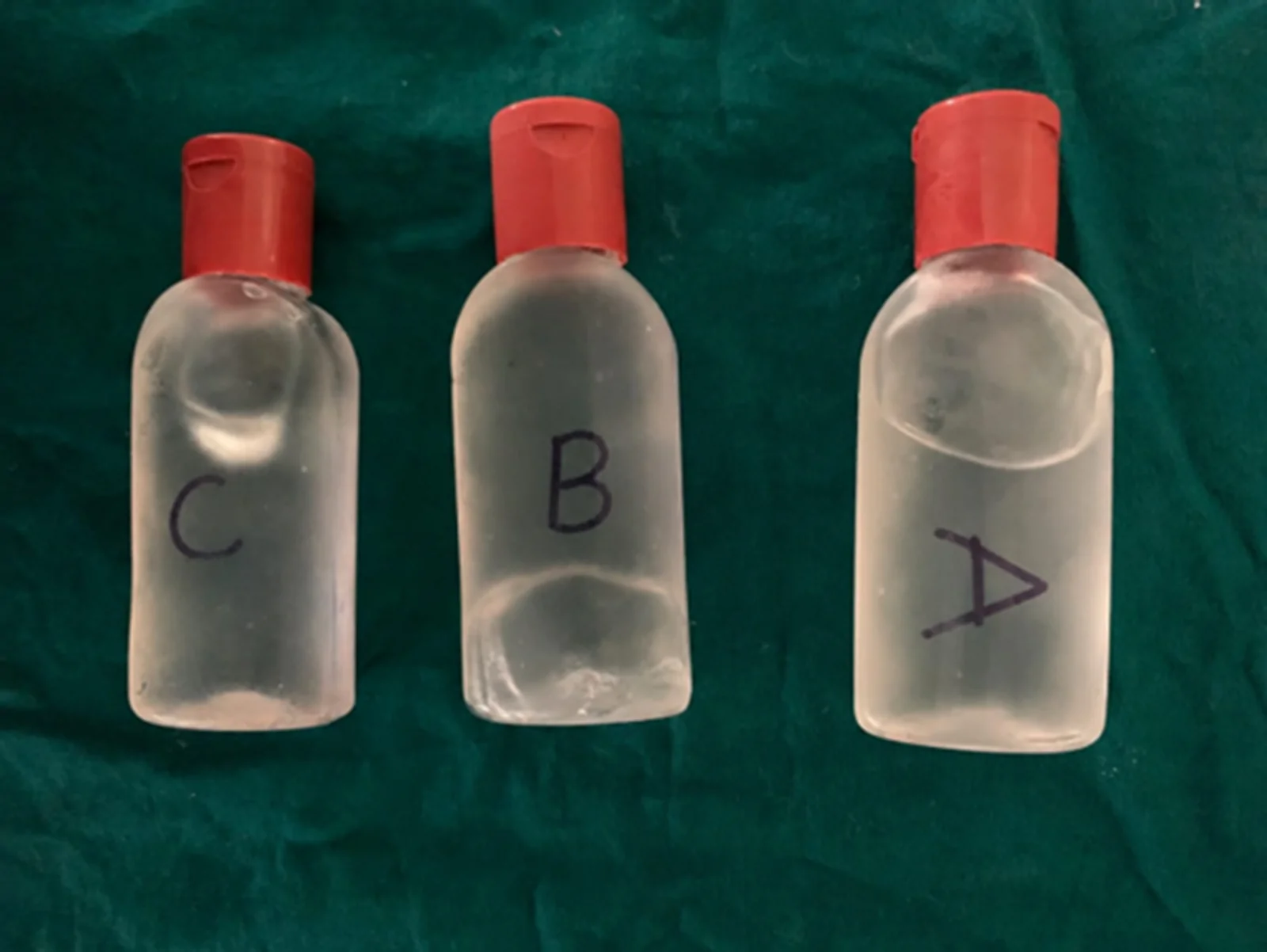
1.2% of RSV, SMV, and Placebo Gel Bottles

The statin powder was taken from Aurobindo Pharmaceutical Company, and the formulation and evaluation of Statin gel for periodontal use and local administration was carried out in KP Labs, Kothapet, Hyderabad. 2.5 g of carboxymethylcellulose was added to 100 g of water gradually while stirring continuously to achieve a gel-like consistency. After this mixture was prepared, 1.2 g of SMV/RSV was incorporated slowly while maintaining continuous stirring to complete the preparation. (Fig. 1)

Following completion of baseline clinical and microbiological assessments, the selected periodontal sites were randomly allocated in a 1:1:1 ratio to one of the three intervention groups: Group I (SRP + 1.2% rosuvastatin gel), Group II (SRP + 1.2% simvastatin gel), and Group III (SRP + placebo gel). A computer-generated random allocation sequence was prepared by an independent investigator who was not involved in participant recruitment, intervention delivery, or outcome assessment. Randomization was performed using block randomization with a block size of six to ensure balanced allocation across the three study groups.

The allocation sequence was concealed using sequentially numbered, opaque, sealed envelopes (SNOSE). After completion of scaling and root planing and baseline measurements, the treating clinician opened the next envelope in sequence to determine the assigned intervention. The investigator responsible for recording clinical and microbiological outcomes remained blinded to the treatment allocation throughout the study.

### Primary outcome

The primary outcome of the study was the change in probing pocket depth (PPD) from baseline to 3 months following treatment. PPD was measured in millimetres using a University of North Carolina (UNC-15) periodontal probe at baseline, 6 weeks, and 3 months. The primary comparison was the difference in mean PPD reduction among the three treatment groups.

### Secondary outcomes

The secondary outcomes included:

Change in clinical attachment level (CAL) from baseline to 3 months.

Change in Modified Gingival Index (MGI) scores at baseline, 6 weeks, and 3 months.

Change in Sulcus Bleeding Index (SBI) scores at baseline, 6 weeks, and 3 months.

Change in Plaque Index (PI) scores at baseline, 6 weeks, and 3 months.

Change in the microbiological levels of *Porphyromonas gingivalis* and *Aggregatibacter actinomycetemcomitans*, assessed by real-time polymerase chain reaction (PCR) using cycle threshold (Ct) values at baseline and 6 weeks.

Incidence of adverse events, allergic reactions, or other treatment-related complications observed during the study period.

All clinical measurements were recorded by a calibrated examiner using standardized periodontal assessment methods, and microbiological analyses were performed under uniform laboratory conditions.

### Sample collection, preparation & storage

**Fig. 2:**
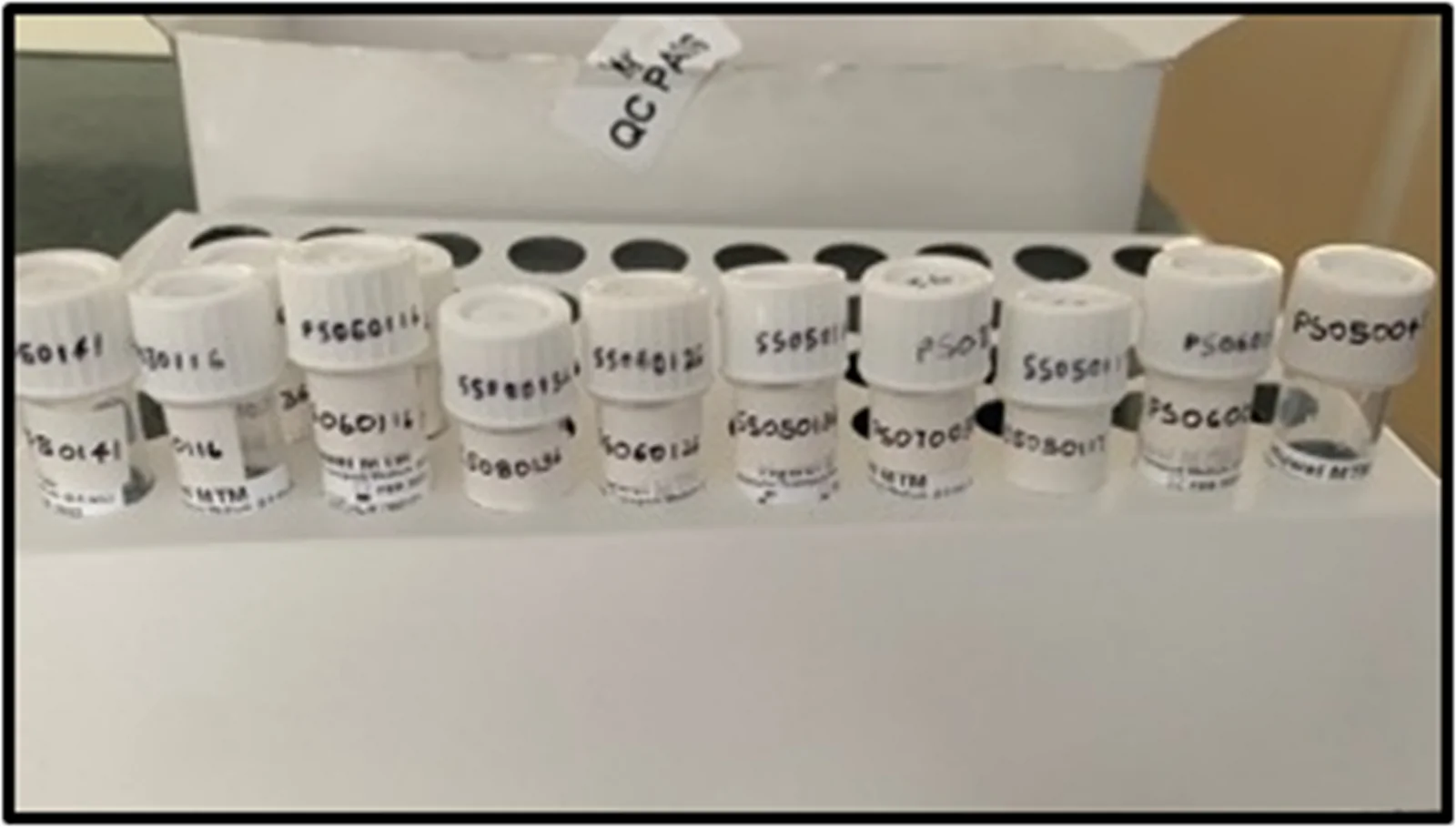
Plaque samples collected in the transport media

Baseline readings were taken to read values of PI, MGI, SBI, PPD, and CAL with the University of North Carolina probe and subgingival plaque was taken using the area-specific Gracey curette and moved into a vial of Huwel Molecular Transport Media (MTM). All the samples were stored at -20 °C till the PCR performed. (Fig. 2)

### Application of the gel

A plaque sample was collected, and scaling/root planing was carried out. Local drug delivery was performed manually using a blunt-cannula syringe, and the prepared placebo, 1.2% RSV gel, or 1.2% SMV gel was administered into the periodontal pocket at a dose of 0.1 mL.

**Fig. 3:**
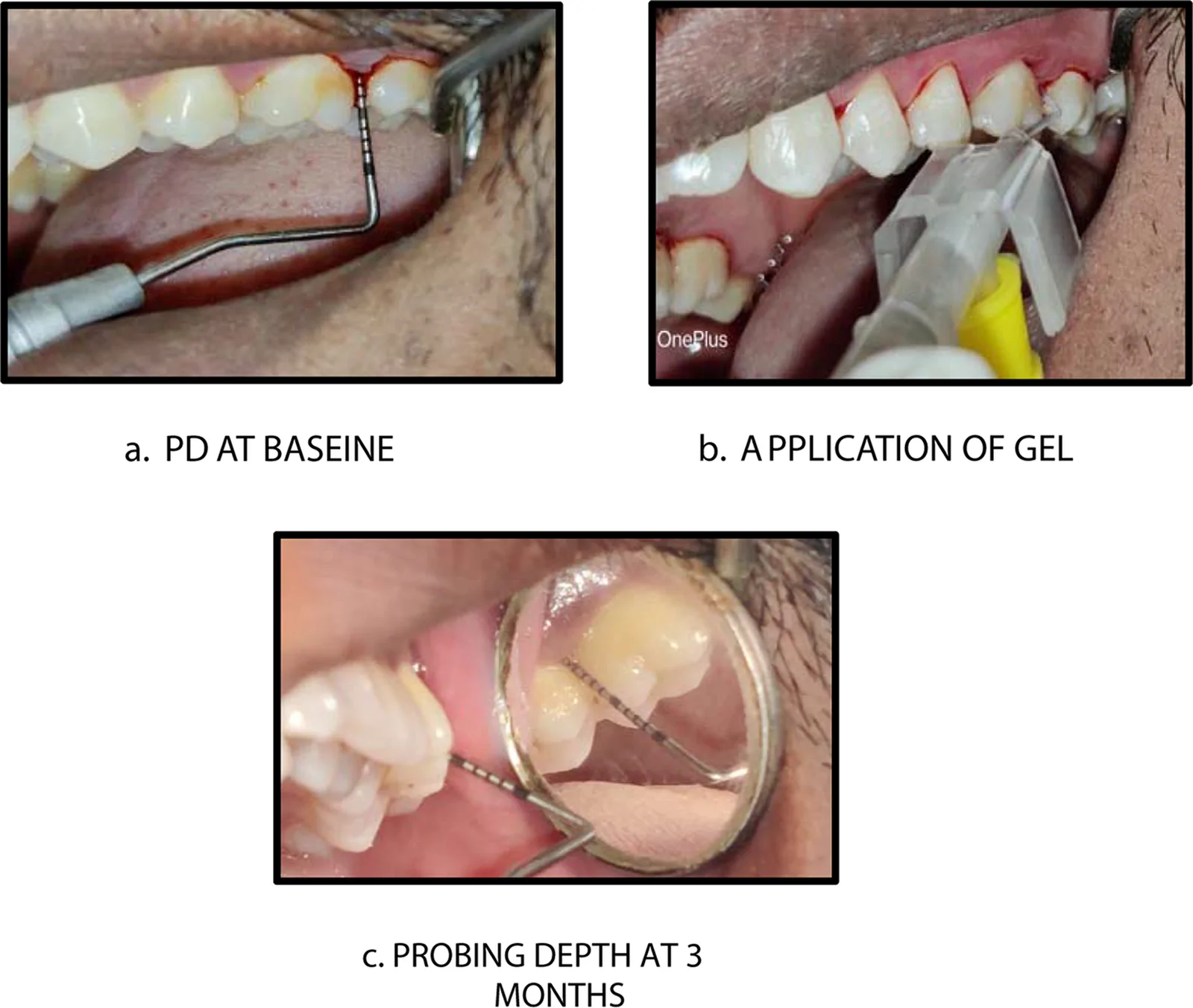
Rosuvastain group

**Fig. 4:**
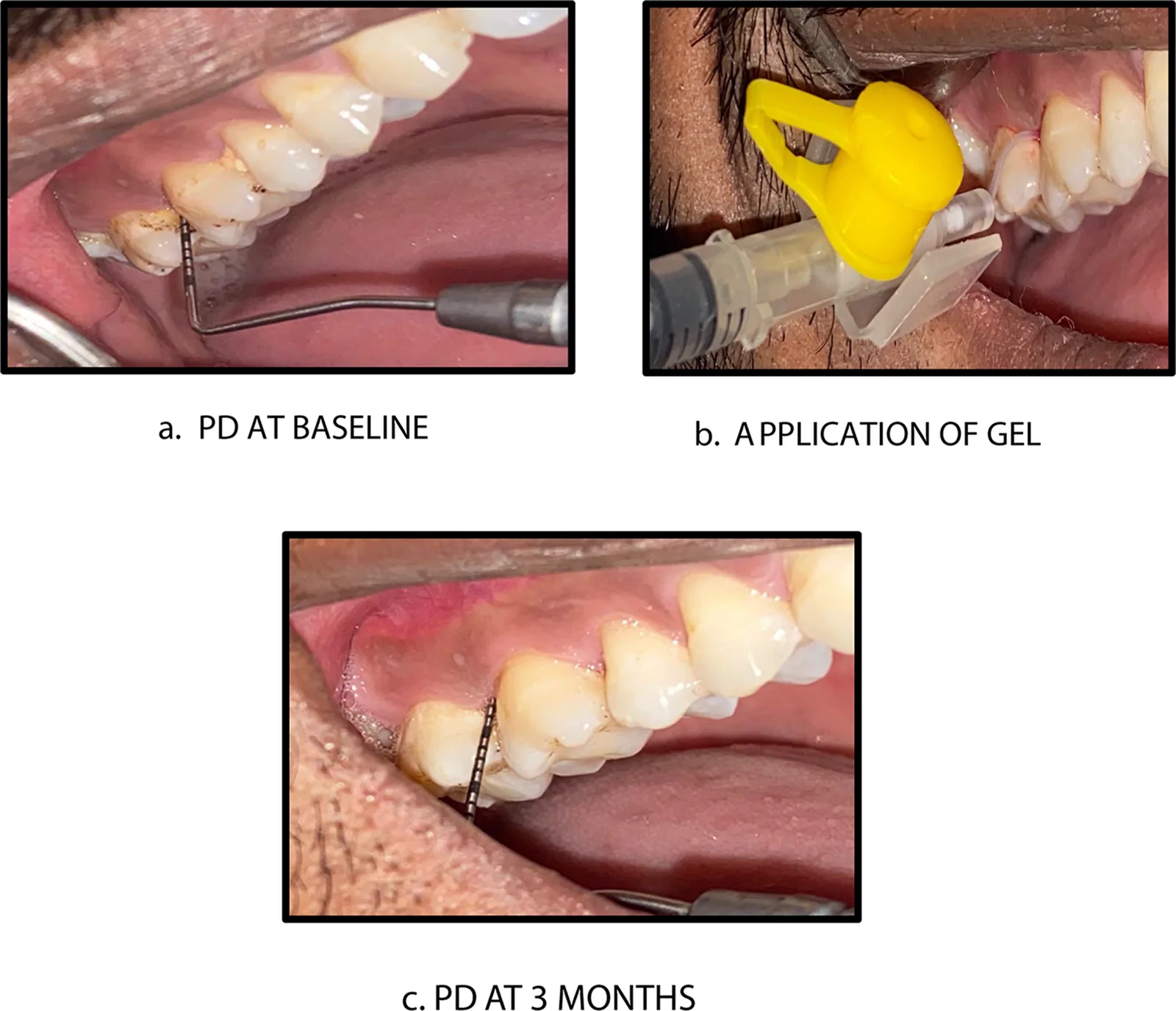
Simvastatin group

**Fig. 5:**
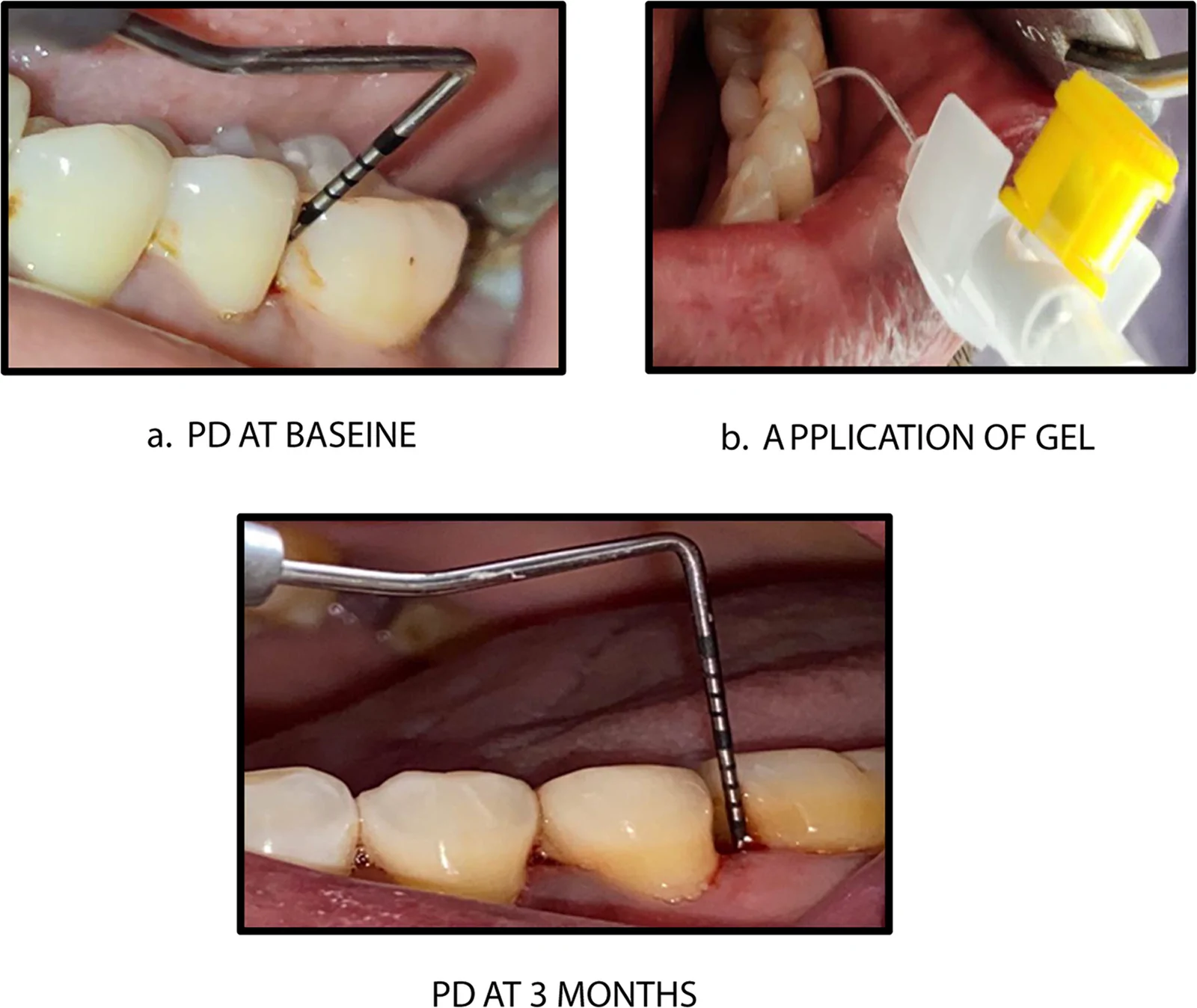
Placebo group

After delivery, the gel became more viscous as carboxymethylcellulose absorbs water from the surrounding GCF and binds to the mucin layer of the pocket epithelium. This hydration causes polymer chain expansion and physical entanglement, raising local viscosity, and fills the pockets, thus eliminating the need for a periodontal dressing (Viglianisi et al. 2023). After LDD, patients were instructed to avoid sticky hard food products as well as toothbrush and interdental aids around the treated areas for a week to avoid extrusion of the gel from the pocket (Pradeep and Thorat 2010). The postoperative instructions were the same for all the patients, and the use of mouthwashes or antibiotics was not indicated after therapy. (Fig. 3, 4, 5)

## Results

In this study, 60 sites were analyzed among patients aged 25 to 55 years. There were no dropouts in the study. Clinical parameters were evaluated at baseline, 6 weeks, and 3 months. Microbiological assessments were conducted at baseline and 6 weeks. Upon clinical evaluation, none of the patients reported any adverse or allergic reactions. Soft tissue healing was uneventful.

### Statistical analysis

Data were analyzed using SPSS version 20. The Kruskal-Wallis test was used to compare the three groups. However, the pairwise comparison of two groups was done using the Mann-Whitney U test. Kruskal wallis Anova test showed that there was no statistically significant difference between the three groups in both clinical and microbiological parameters at baseline. All three groups showed a statistically significant reduction in all the parameters.

The difference in plaque scores amongst the three groups was not statistically significant at any time interval. Statin groups showed lower MGI, SBI scores, probing depths, and CAL at 6 weeks and 3 months compared to the placebo group, and this difference was statistically significant **(P < = 0.05).** Between the statin groups, Simvastatin showed a greater reduction in MGI and SBI scores and a greater reduction in **probing depth** and **CAL** at 6 weeks and 3 months. However, this difference was not statistically significant **(P = > 0.05).**

**Graph. 1:**
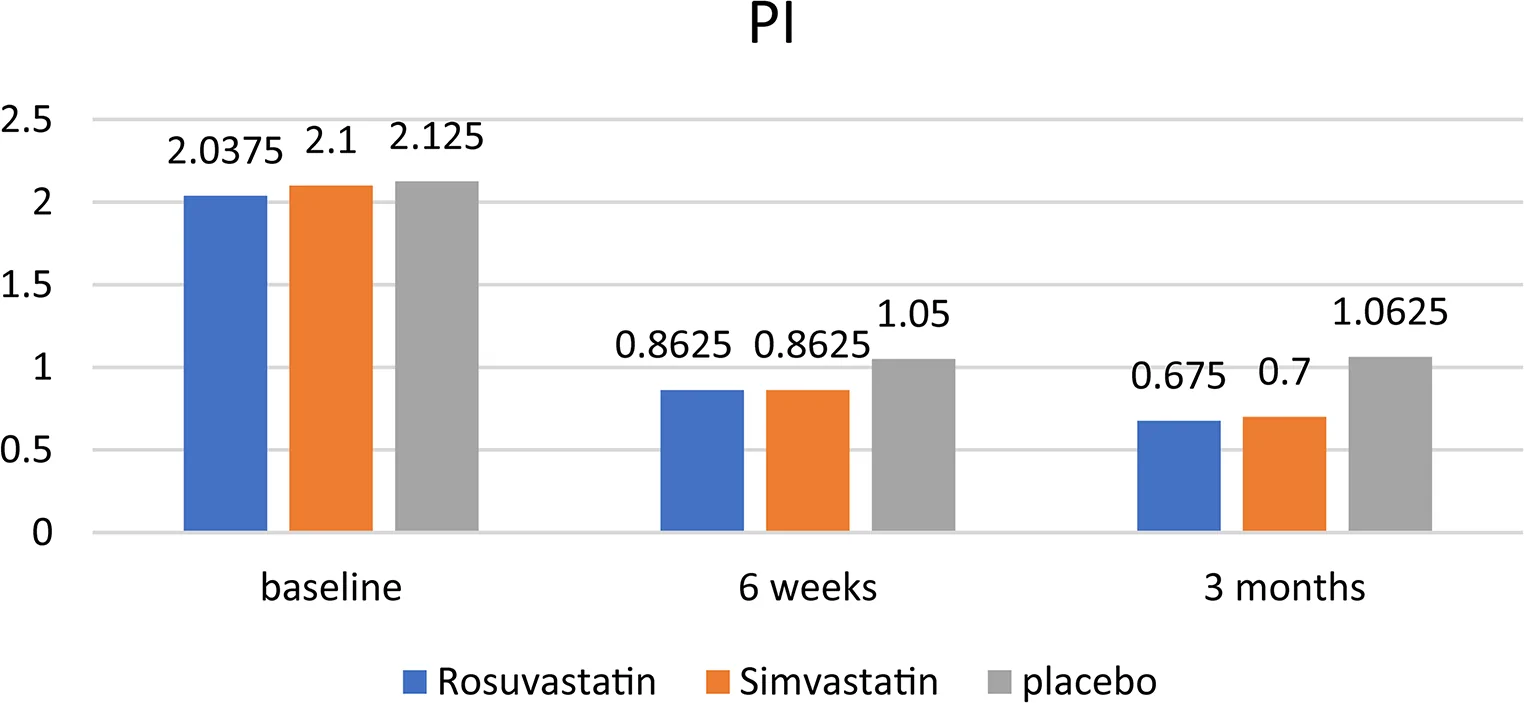
Intergroup comparison of plaque index at baseline, 6 weeks and 3 months

**Graph. 2:**
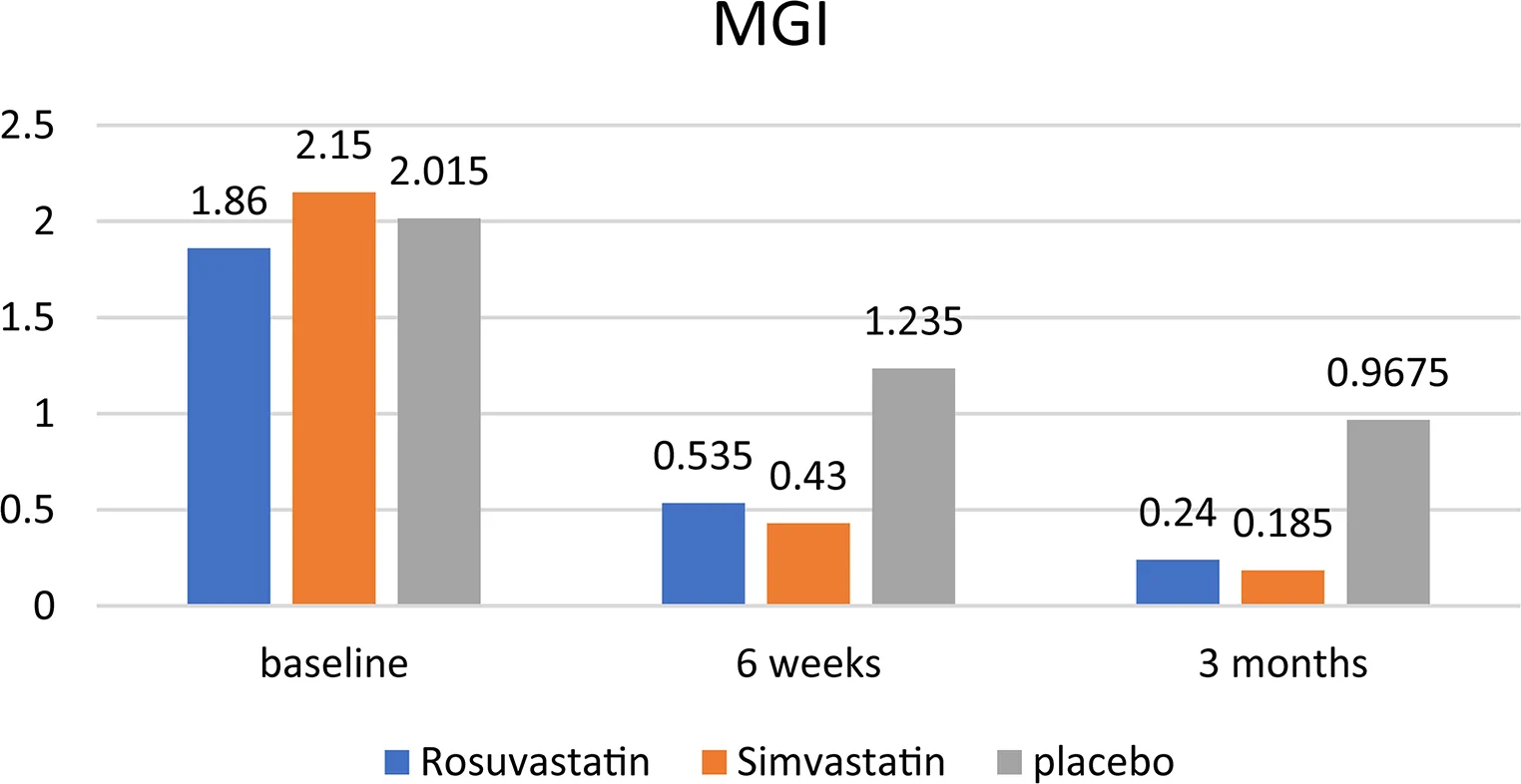
Intergroup comparison of modified gingival index at baseline, 6 weeks and 3 months

**Graph. 3:**
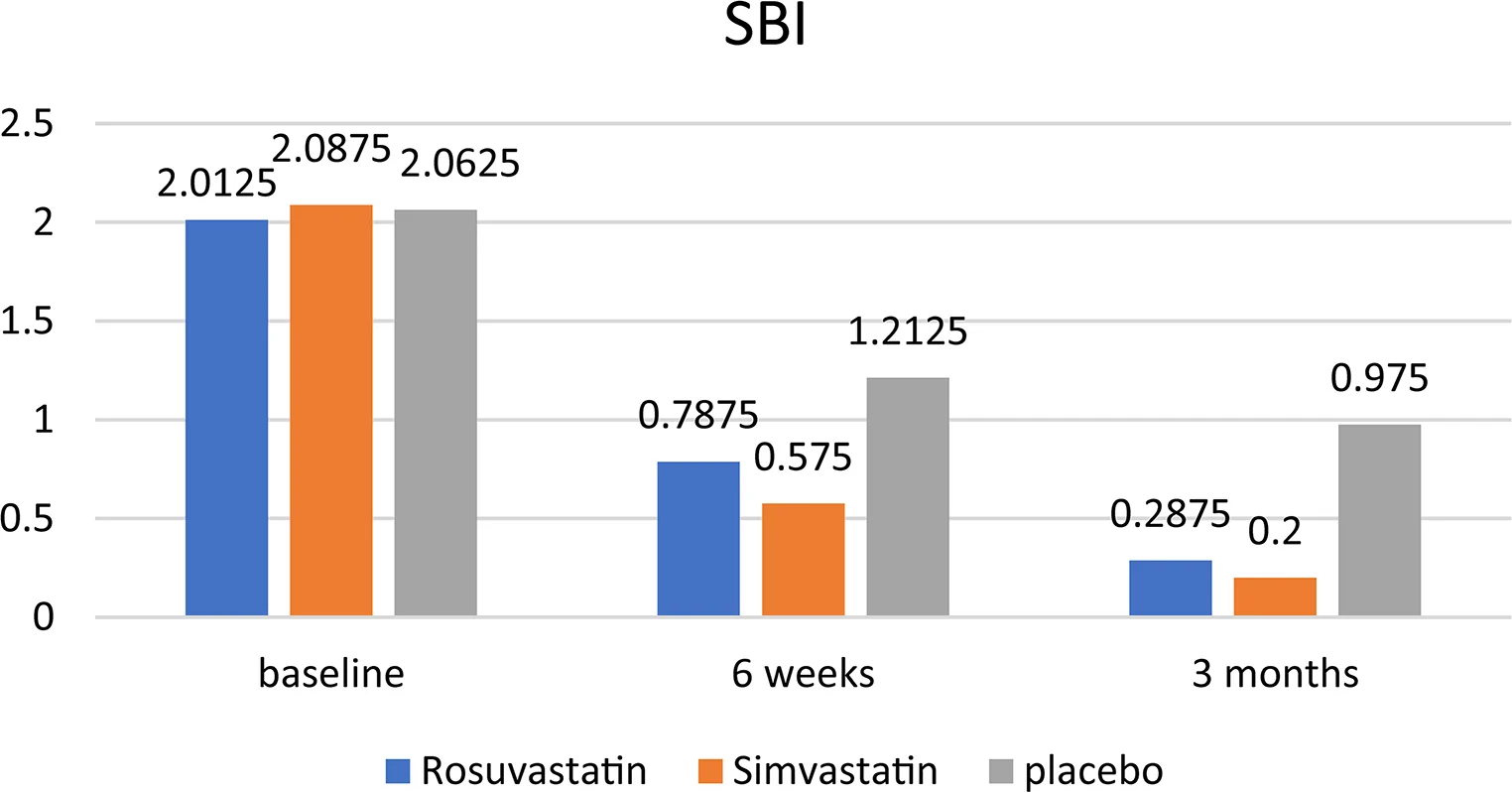
Intergroup comparison of sulcus bleeding at baseline, 6 weeks and 3 months

**Graph. 4:**
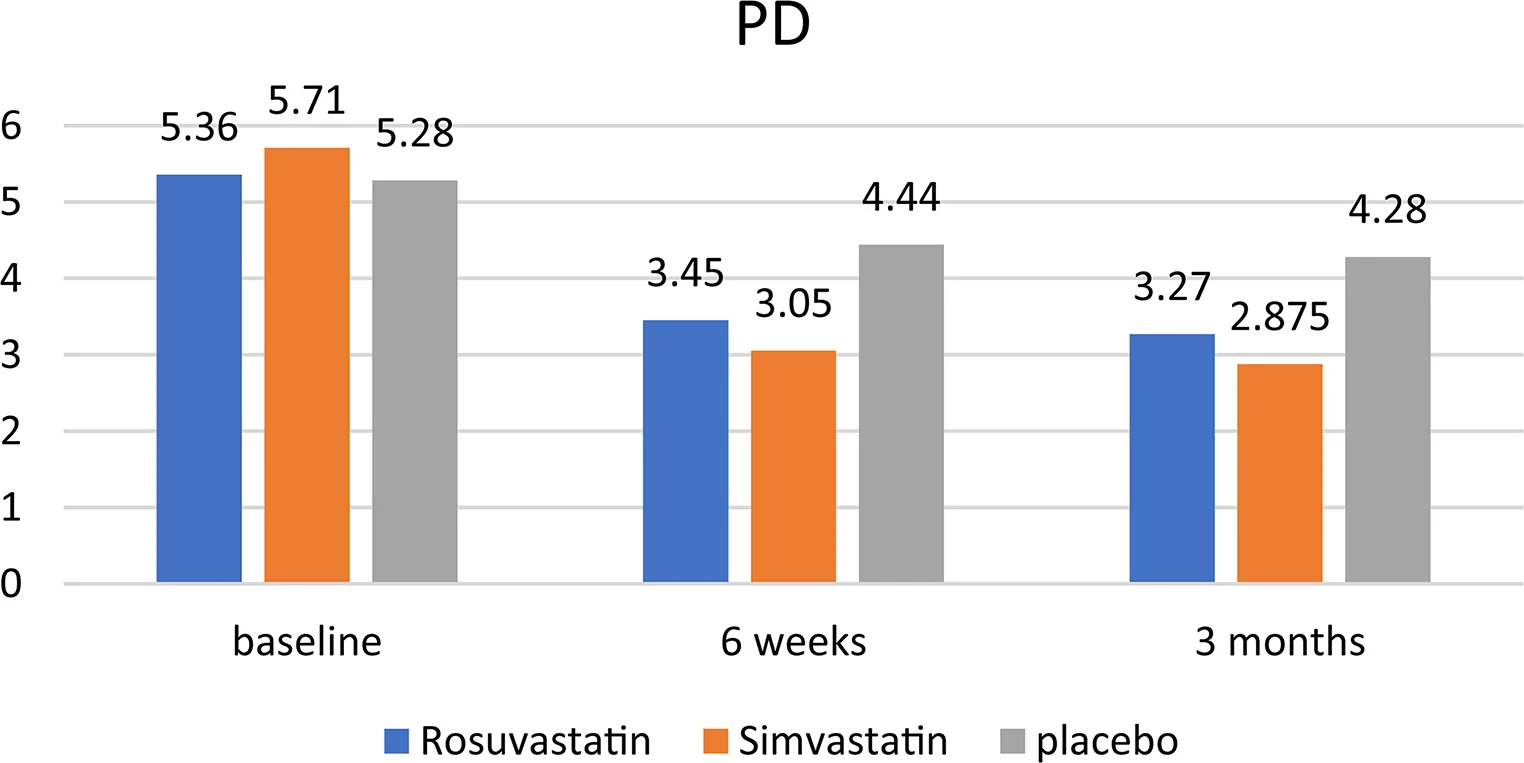
Intergroup comparison of Probing depth at baseline, 6 weeks and 3 months

**Graph. 5:**
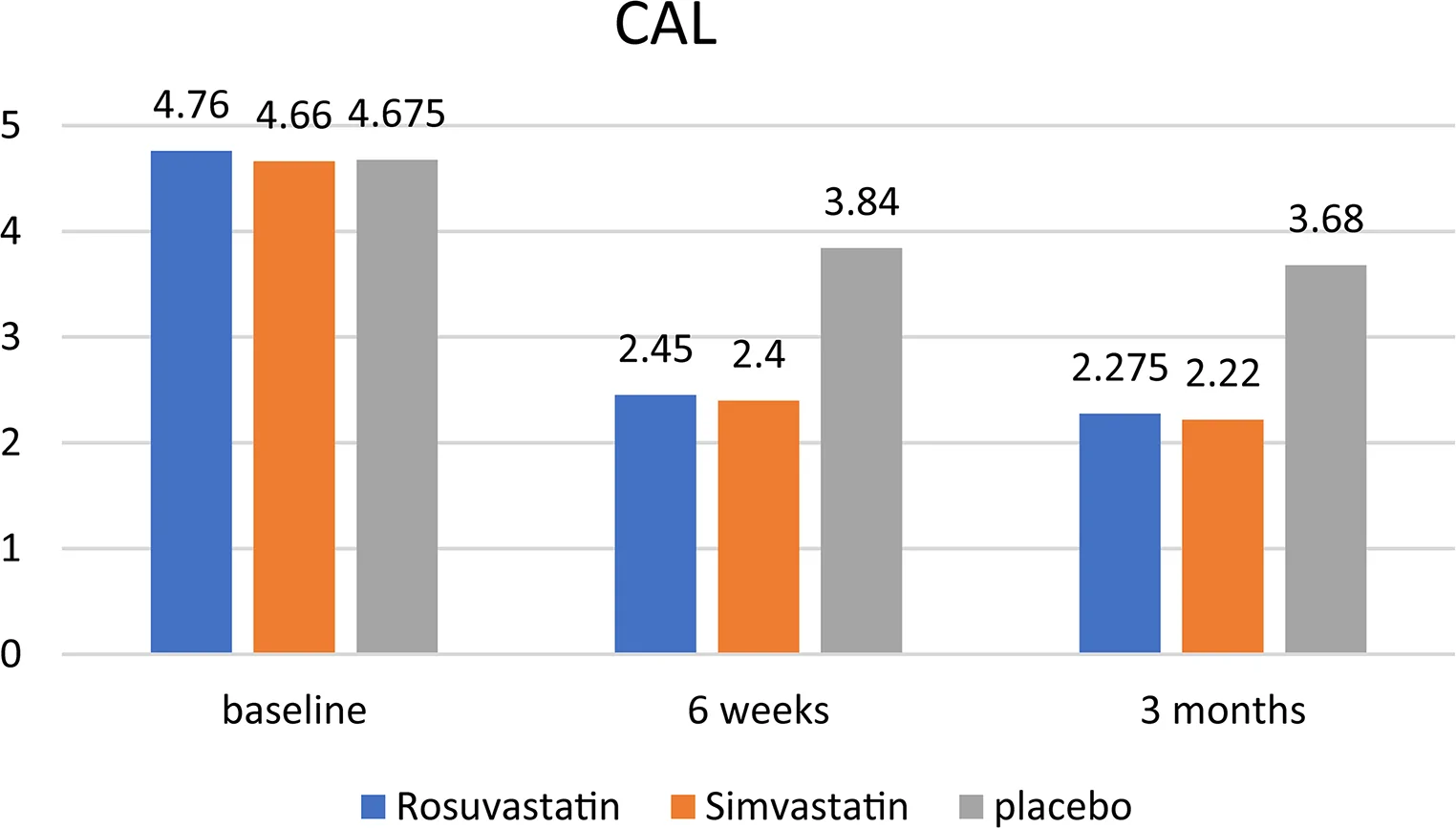
Intergroup comparison of Clinical attachment level at baseline, 6 weeks and 3 months

**Graph. 6:**
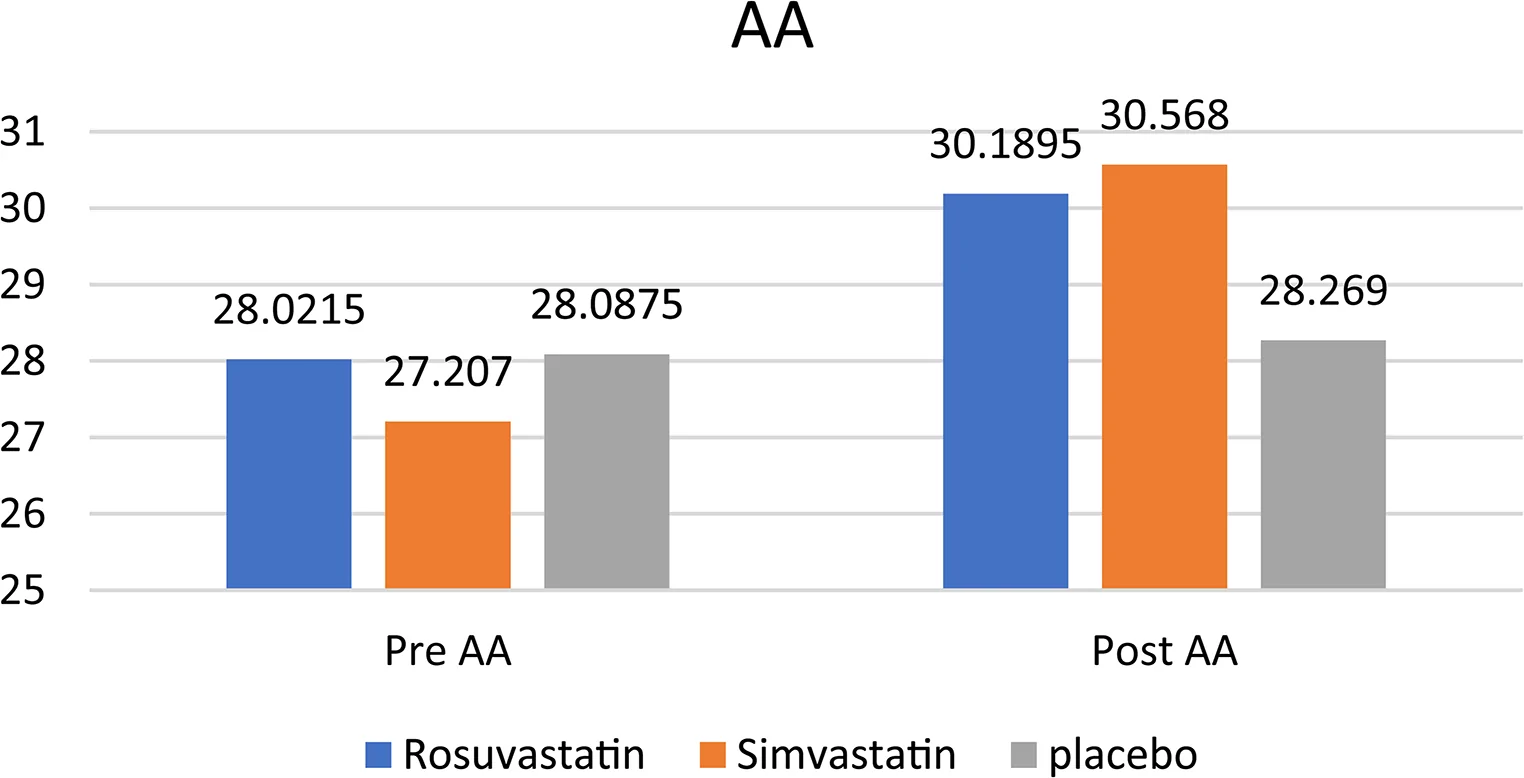
Intergroup comparison of Aggregatibacter actinomycetemcomitans at baseline and 6 weeks

**Graph. 7:**
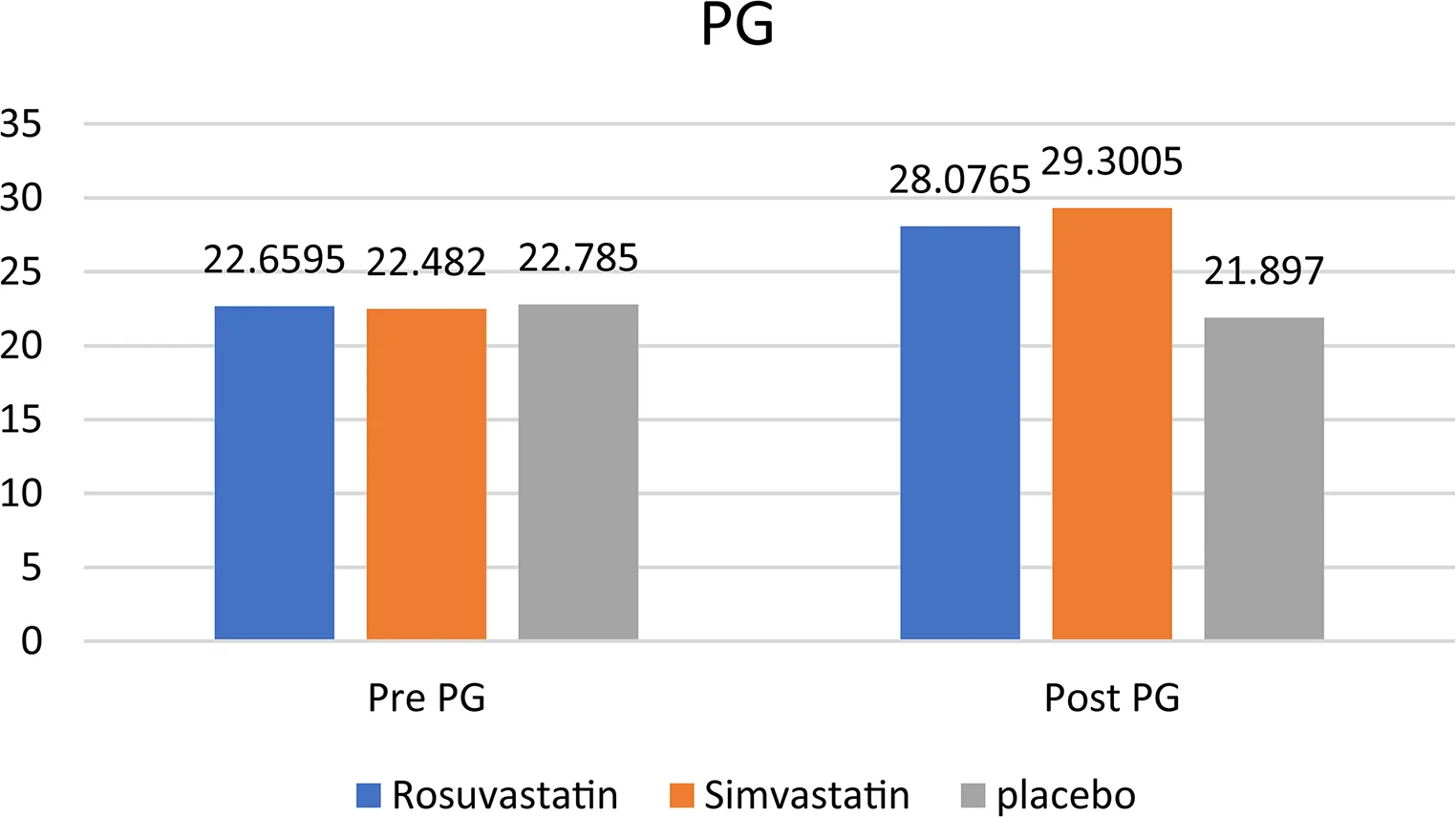
Intergroup comparison of Porphyromonas gingivalis at baseline and 6 weeks

The statin groups also showed a greater **Ct** value for **Aa and Pg** at 6 weeks, and this difference was statistically significant **(P < = 0.05). Ct** values are inversely proportional to the amount of bacterial DNA present. Between the statin groups, Simvastatin showed a greater reduction in Aa and Pg at 6 weeks. However, this difference was not statistically significant **(P = > 0.05) **(Graph. 1, 2, 3, 4, 5, 6, 7).

## Discussion

Local delivery of the drug is a minimally invasive method of reducing the bacterial load. Due to various non-lipidic effects of statins such as antibacterial, anti-inflammatory, immunomodulatory, etc., statins are used as a local drug delivery and host-modulating agent in periodontics (Purushotham et al. 2015). **A R Pradeep et al.** and **S S Martande et al.** conducted studies comparing 1.2% Rosuvastatin with 1.2% Atorvastatin gel and 1.2% Simvastatin with 1.2% Atorvastatin gel, respectively. ^**9,10**^ **Ramezani A et al.** conducted research comparing the antimicrobial effect of Atorvastatin and nano Atorvastatin mouthwash on Aggregatibacter actinomycetemcomitans using a micro broth dilution assay; the result revealed that the MIC of atorvastatin and nano atorvastatin was 0.039 and 0.002 µg/mL, respectively. This study suggested that the compounds could be useful as adjunctive treatment of periodontitis (Ramezani et al. 2026).

To the best of our knowledge based on the literature surveyed, no studies are comparing the clinical efficacy and antimicrobial properties of 1.2% SMV and 1.2% RSV gels in patients with chronic periodontitis. Therefore, the current study aimed to evaluate the clinical and antimicrobial effects of simvastatin and rosuvastatin gels. In the present study, the efficacy of 1.2% RSV and 1.2% SMV was compared based on clinical and microbiological parameters.

Gram-negative bacteria such as *Aggregatibacter actinomycetemcomitans*, *Porphyromonas gingivalis*, *Tannerella forsythia*, and *Treponema denticola* are significantly predominant in the periodontal pocket and are associated with the advancement of periodontal disease (Kulkarni et al. 2018). Kulkarini PG, in his study, detected Pg in 60% of patients having pocket depth upto 5 mm and in 93.33% of patients having more than 5 mm pocket depth (Kulkarni et al. 2018). In a study by **Joshi VM**, Aa was found in 60% of patients having chronic periodontitis (Joshi et al. 2016). Gohler A et al., conducted a comparative study on the microbiological samples collected from two oral habitats (Tongue + subgingival pocket) and found that increased relative abundances of P. gingivalis, A. actinomycetemcomitans, and F. nucleatum were linked to increased levels of PD or CAL (Göhler et al. 2018). Hence, in this study, it was decided to check the Aa and Pg levels in periodontal pockets before and after the application of statins.

To identify and detect bacteria in the present study, polymerase chain reaction (PCR) was used. PCR demonstrates greater sensitivity and specificity in detecting bacteria compared to bacterial culture (Kulkarni et al. 2018). Microorganisms were detected based on the cycle threshold (Ct) value. The number of cycles needed for the fluorescent signal to cross the threshold above background signal is defined as the Ct. The lower the concentration of target nucleic acid in the sample, the higher the Ct.

In this study, a statistically significant reduction in plaque scores was observed from baseline to the end of the study (3 months) in each of the three groups. However, the difference among the three groups was not statistically significant, indicating no difference in oral hygiene status across all groups throughout the study period.

In a double-blind randomized clinical trial, Lobene et al. sought to determine the relationship between the Modified Gingival Index (MGI) and gingival inflammatory indices that involve a bleeding component. In the present study, MGI was used because it has been proven comparable to indices traditionally used in gingivitis studies (Lobene 1986) while avoiding invasive procedures that could disrupt soft tissue or plaque in the gingival region.

In the present study, all clinical parameters significantly improved in the placebo group. A decrease in the levels of *A. actinomycetemcomitans* (*A.a.*) and *P. gingivalis* (*P.g.*) was also observed in the placebo group; however, this did not reach statistical significance. This is in line with the findings of Pradeep et al., who also reported significant improvement in clinical parameters within the placebo group (Pradeep et al. 2015). 

In this study, SMV and RSV showed statistically significant improvement compared to placebo in all clinical parameters from baseline to three months. This shows the anti-inflammatory effect of SMV and RSV. This is in accordance with previous studies done by other researchers (Agarwal et al. 2016, Pradeep et al. 2015, Rath et al. 2012, Pradeep et al. 2013, Grover et al. 2016, Stalker et al. 2001, Saba et al. 2025, Pietrzko et al. 2025). 

Statins exert anti-inflammatory effects by both decreasing the levels of pro-inflammatory cytokines (IL-6, IL-8) and increasing the levels of anti-inflammatory cytokines (IL-4, IL-5, IL-10 (Grover et al. 2016, Saba et al. 2025, Cicek Ari et al. 2016). They are also involved in the production of pro-resolving molecules (Spite and Serhan 2010) such as lipoxins, resolvins, maresins and protectins; however, further investigations are required to confirm this. Statins have also been shown to have an effect on Lymphocyte Function Associated Antigen-1 (LFA-1). Intercellular Adhesion Molecule-1(ICAM-1) regulates lymphocyte function-associated antigen-1 (LFA-1) dependent neutrophil transmigration and recruitment to the inflammation site. Studies have also found that statins inhibit LFA-1, therefore having a role in ‘host modulation’(Catherine Petit et al. 2019, Hajishengallis and Sahingur 2014). Statins have also been found to have an inhibitory effect on Matrix metalloproteinases (MMP)-1, 8, and 9 and Nitric Oxide Synthase (NOS) (Catherine Petit et al. 2019, Poston et al. 2016).

Statistically, a greater reduction in *A. actinomycetemcomitans* (*A.a.*) and *P. gingivalis* (*P.g.*) scores was observed with Simvastatin and Rosuvastatin than with placebo in our study. This is likely because these statins possess antibacterial effects, which aligns with findings from previous studies (Kamińska et al. 2019, Emani et al. 2014). 

In their review, Petit et al. detailed the pleiotropic effects of statins. A reduction in reactive oxygen species (ROS), an increase in pro-resolving molecules, and the inhibition of cholesterol synthesis were cited as responsible for the antibacterial properties of statins (Catherine Petit et al. 2019).

In this study, SMV showed greater improvements compared to RSV in all the clinical parameters as well as greater reduction in Aa and Pg levels. However, this was not statistically significant. This could be due to greater anti-bacterial effect of SMV or could be by chance. Hence, further randomized clinical trials with large cohort are required to evaluate the effectiveness of Simvastatin over Rosuvastatin.

## Conclusion

Periodontitis is a chronic inflammatory condition resulting from a dysregulated host immune response to microbes. The application of controlled-release, locally administered antimicrobial agents in conjunction with mechanical therapy yields positive outcomes in managing localized periodontal sites. Statins have been shown to exhibit various pleiotropic effects, including anti-inflammatory, antioxidant, antibacterial, and immunomodulatory properties. These properties make statins promising agents in the treatment of periodontitis. Simvastatin is an older, naturally derived lipophilic drug, whereas rosuvastatin is a newer, synthetic, hydrophilic drug. Therefore, the present study aimed to compare their clinical and antimicrobial activities. Both drugs showed statistically significant improvements in clinical and microbiological parameters compared to placebo. Between the two statins, simvastatin showed better overall results than rosuvastatin; however, the difference between them was not statistically significant.

Further, well-designed long-term interventional studies with a large sample size are required to substantiate the findings of the present study and prove the efficacy of Simvastatin over Rosuvastatin.

**Table:** 

Tests of Normality							
	group	Kolmogorov-Smirnov^a^			Shapiro-Wilk		
		Statistic	df	Sig.	Statistic	df	Sig.
baseline	Rosuvastatin	0.307	17	0.000	0.807	17	0.003
	Simvastatin	0.346	17	0.000	0.761	17	0.001
	placebo	0.328	20	0.000	0.728	20	0.000
6 weeks	Rosuvastatin	0.364	17	0.000	0.784	17	0.001
	Simvastatin	0.388	17	0.000	0.758	17	0.001
	placebo	0.236	20	0.005	0.875	20	0.014
3 months	Rosuvastatin	0.316	17	0.000	0.791	17	0.002
	Simvastatin	0.363	17	0.000	0.701	17	0.000
	placebo	0.184	20	0.074	0.864	20	0.009
baseline	Rosuvastatin	0.221	17	0.027	0.836	17	0.007
	Simvastatin	0.497	17	0.000	0.470	17	0.000
	placebo	0.471	20	0.000	0.436	20	0.000
6 weeks	Rosuvastatin	0.284	17	0.001	0.839	17	0.007
	Simvastatin	0.285	17	0.001	0.824	17	0.004
	placebo	0.233	20	0.006	0.884	20	0.021
3 months	Rosuvastatin	0.302	17	0.000	0.578	17	0.000
	Simvastatin	0.257	17	0.004	0.799	17	0.002
	placebo	0.405	20	0.000	0.725	20	0.000
baseline	Rosuvastatin	0.264	17	0.003	0.896	17	0.058
	Simvastatin	0.493	17	0.000	0.498	17	0.000
	placebo	0.415	20	0.000	0.633	20	0.000
6 weeks	Rosuvastatin	0.391	17	0.000	0.688	17	0.000
	Simvastatin	0.309	17	0.000	0.846	17	0.009
	placebo	0.370	20	0.000	0.792	20	0.001
3 months	Rosuvastatin	0.299	17	0.000	0.771	17	0.001
	Simvastatin	0.296	17	0.000	0.782	17	0.001
	placebo	0.281	20	0.000	0.871	20	0.012
baseline	Rosuvastatin	0.170	17	0.200^*^	0.889	17	0.044
	Simvastatin	0.225	17	0.023	0.872	17	0.024
	placebo	0.218	20	0.014	0.889	20	0.026
6 weeks	Rosuvastatin	0.214	17	0.037	0.899	17	0.066
	Simvastatin	0.214	17	0.038	0.868	17	0.020
	placebo	0.222	20	0.011	0.855	20	0.007
3 months	Rosuvastatin	0.290	17	0.001	0.879	17	0.031
	Simvastatin	0.186	17	0.122	0.879	17	0.031
	placebo	0.337	20	0.000	0.821	20	0.002
baseline	Rosuvastatin	0.220	17	0.029	0.881	17	0.033
	Simvastatin	0.244	17	0.008	0.826	17	0.005
	placebo	0.182	20	0.080	0.903	20	0.047
6 weeks	Rosuvastatin	0.206	17	0.054	0.890	17	0.045
	Simvastatin	0.242	17	0.009	0.871	17	0.023
	placebo	0.163	20	0.171	0.885	20	0.022
3 months	Rosuvastatin	0.205	17	0.056	0.847	17	0.010
	Simvastatin	0.255	17	0.005	0.851	17	0.011
	placebo	0.230	20	0.007	0.834	20	0.003
AA pre	Rosuvastatin	0.308	17	0.000	0.671	17	0.000
	Simvastatin	0.121	17	0.200^*^	0.974	17	0.884
	placebo	0.106	20	0.200^*^	0.958	20	0.496
Pg pre	Rosuvastatin	0.196	17	0.083	0.867	17	0.020
	Simvastatin	0.140	17	0.200^*^	0.963	17	0.696
	placebo	0.176	20	0.106	0.924	20	0.117
AA post	Rosuvastatin	0.160	17	0.200^*^	0.869	17	0.021
	Simvastatin	0.191	17	0.098	0.888	17	0.043
	placebo	0.163	20	0.172	0.969	20	0.737
Pg post	Rosuvastatin	0.236	17	0.013	0.726	17	0.000
	Simvastatin	0.111	17	0.200^*^	0.961	17	0.651
	placebo	0.177	20	0.101	0.878	20	0.016
*. This is a lower bound of the true significance.							

a. Lilliefors Significance Correction

**Table:** 

			N	Mean	Std. Deviation	Chi square value	P value
PI	baseline	Rosuvastatin	20	2.0375	0.38281	0.98	0.61
		Simvastatin	20	2.1000	0.46169		
		placebo	20	2.1250	0.40148		
	6 weeks	Rosuvastatin	20	0.8625	0.45505	0.43	0.80
		Simvastatin	20	0.8625	0.31908		
		placebo	20	1.0500	0.55961		
	3 months	Rosuvastatin	20	0.6750	0.50719	4.04	0.13
		Simvastatin	20	0.7000	0.36814		
		placebo	20	1.0625	0.72943		
MGI	baseline	Rosuvastatin	20	1.8600	0.72286	4.4	0.10
		Simvastatin	20	2.1500	0.36635		
		placebo	20	2.0150	0.27961		
	6 weeks	Rosuvastatin	20	0.5350	0.48480	25.34	0.000 HS
		Simvastatin	20	0.4300	0.37989		
		placebo	20	1.2350	0.37735		
	3 months	Rosuvastatin	20	0.1850	0.30136	36.04	0.000 HS
		Simvastatin	20	0.2400	0.20876		
		placebo	20	0.9675	0.23299		
SBI	baseline	Rosuvastatin	20	2.0125	0.79668	4.88	0.08
		Simvastatin	20	2.0875	0.21877		
		placebo	20	2.0625	0.37936		
	6 weeks	Rosuvastatin	20	0.7875	0.45360	14.3	0.001 HS
		Simvastatin	20	0.5750	0.38984		
		placebo	20	1.2125	0.48174		
	3 months	Rosuvastatin	20	0.2875	0.36522	31.77	0.000 HS
		Simvastatin	20	0.2000	0.19194		
		placebo	20	0.9750	0.32343		
PD	baseline	Rosuvastatin	20	5.3600	0.33623	8.83	0.01 S
		Simvastatin	20	5.7100	0.52103		
		placebo	20	5.2800	0.45259		
	6 weeks	Rosuvastatin	20	3.0500	0.37205	41.73	0.000 HS
		Simvastatin	20	3.4500	0.43830		
		placebo	20	4.4400	0.45003		
	3 months	Rosuvastatin	20	2.8750	0.22682	43.22	0.000 HS
		Simvastatin	20	3.2700	0.46009		
		placebo	20	4.2800	0.44081		
CAL	baseline	Rosuvastatin	20	4.7600	0.97300	0.01	0.99
		Simvastatin	20	4.6600	0.70815		
		placebo	20	4.6750	0.84160		
	6 weeks	Rosuvastatin	20	2.4500	1.15690	23.41	0.000 HS
		Simvastatin	20	2.4000	0.72184		
		placebo	20	3.8400	0.75422		
	3 months	Rosuvastatin	20	2.2750	1.09009	23.44	0.000 HS
		Simvastatin	20	2.2200	0.70755		
		placebo	20	3.6800	0.71126		
Pre	AA pre	Rosuvastatin	20	28.0215	3.21061	5.54	0.06
		Simvastatin	20	27.2070	1.59860		
		placebo	20	28.0875	2.02939		
	Pg pre	Rosuvastatin	20	22.6595	6.80207	0.01	0.99
		Simvastatin	20	22.4820	4.89192		
		placebo	20	22.7850	6.57033		
Post	AA post	Rosuvastatin	20	30.1895	2.69132	11.71	0.003 HS
		Simvastatin	20	30.5680	1.56882		
		placebo	20	28.2690	1.98275		
	Pg post	Rosuvastatin	20	28.0765	4.73871	14.04	0.001 HS
		Simvastatin	20	29.3005	2.95900		
		placebo	20	21.8970	6.16020		

Statistical test applied: Kruskal wallis Anova

**Table:** Pair wise comparisons (Rosuvastatin vs. Simvastatin)

		Z	Asymp. Sig. (2-tailed)
PI	baseline	− 0.572	0.567
	6 weeks	− 0.175	0.861
	3 months	− 0.948	0.343
MGI	baseline	-1.815	0.070
	6 weeks	− 0.512	0.609
	3 months	-1.361	0.174
SBI	baseline	-1.934	0.053
	6 weeks	-1.637	0.102
	3 months	− 0.131	0.896
PD	baseline	-2.386	0.067
	6 weeks	-2.891	0.004 HS
	3 months	-3.246	0.001 HS
CAL	baseline	− 0.068	0.946
	6 weeks	− 0.652	0.515
	3 months	− 0.667	0.505
Pre	AA pre	-2.408	0.066 S
	Pg pre	− 0.054	0.957
Post	AA post	− 0.203	0.839
	Pg post	-0.867	0.398

Statistical test applied: Mann Whitney U test; HS – Highly significant at *p* < 0.01; S – Significant at *p* < 0.05

**Table:** Pair wise comparisons (Rosuvastatin vs. Placebo)

		Z	Asymp. Sig. (2-tailed)
PI	baseline	-1.018	0.309
	6 weeks	− 0.575	0.566
	3 months	-1.758	0.079
MGI	baseline	-1.275	0.202
	6 weeks	-3.984	0.000 HS
	3 months	-4.978	0.000 HS
SBI	baseline	-1.638	0.101
	6 weeks	-2.046	0.041 S
	3 months	-4.451	0.000 HS
PD	baseline	− 0.472	0.637
	6 weeks	-5.360	0.000 HS
	3 months	-5.490	0.000 HS
CAL	baseline	− 0.095	0.924
	6 weeks	-3.404	0.001 HS
	3 months	-3.457	0.001 HS
Pre	AA pre	− 0.757	0.449
	Pg pre	− 0.135	0.892
Post	AA post	-2.50	0.012 S
	Pg post	-2.84	0.004 HS

Statistical test applied: Mann Whitney U test; HS – Highly significant at *p* < 0.01; S – Significant at *p* < 0.05

**Table:** Pair wise comparisons (Simvastatin vs. Placebo)

		Z	Asymp. Sig. (2-tailed)
PI	baseline	− 0.334	0.738
	6 weeks	− 0.517	0.605
	3 months	-1.481	0.139
MGI	baseline	-1.344	0.179
	6 weeks	-4.641	0.000 HS
	3 months	-5.303	0.000 HS
SBI	baseline	− 0.266	0.791
	6 weeks	-3.819	0.000 HS
	3 months	-5.242	0.000 HS
PD	baseline	-2.687	0.007 HS
	6 weeks	-5.282	0.000 HS
	3 months	-5.225	0.000 HS
CAL	baseline	− 0.055	0.956
	6 weeks	-4.948	0.000 HS
	3 months	-5.009	0.000 HS
Pre	AA pre	-1.380	0.168
	Pg pre	− 0.068	0.946
Post	AA post	-3.35	0.001 HS
	Pg post	-3.47	0.001 HS

Statistical test applied: Mann Whitney U test; HS – Highly significant at *p* < 0.01; S – Significant at *p* < 0.05

## Data Availability

No datasets were generated or analysed during the current study.
